# The Scottish Syllabus

**Published:** 1921-08-13

**Authors:** 


### THE SCOTTISH SYLLABUS.

The Syllabus of Lectures and Demonstrations for Education and Training in General Nursing issued by the General Nursing Council for Scotland challenges comparison at almost every point with that issued by the English Nursing Council. To be frank, it is nothing like so original. It misses some of the essential points which have been generally admired in its forerunner, and it enters into considerable, perhaps unnecessary, detail in others. Clearly, however, in substance it bespeaks an equally high standard of nursing education, and those who contend that the English Council have imposed a curriculum impossible to be observed will find nothing to support them in the course laid down for Scottish nurses.

In one particular, that of public-health nursing, there is a striking and, in our judgment, a regrettable omission. However briefly the claims of public-health work on the nursing profession are dealt with, it is of the highest importance that they should early be brought to the notice of the probationer and kept in view during her training. Like the English, the Scottish syllabus distinguishes two widely different stages in the instruction of nurses. The first period lasts eighteen months, or less, and comprises teaching in elementary anatomy and physiology, hygiene, elementary theory and practice of nursing, dietetics and cooking. The second period comprises instruction in bacteriology, medical nursing, surgical nursing, gynæcology, and venereal diseases.

Both the English and the Scottish syllabuses lay great stress on the importance of lectures and demonstrations given by fully-trained nurses—*i.e.*, matrons, sister-tutors, and ward sisters. We fail, however, to find in the Scottish course any indication of the means to be adopted to make quite certain that no probationer has missed any point in the necessary practical part of her work. Doubtless it has been thought best to leave matrons to adopt their own system. But that suggested in the English syllabus is so well planned and well-nigh certain in its results that it is likely to be widely used wherever it comes under notice.

